# Efficacy and safety of warm acupuncture combined with Western medicine for sciatica: A protocol for systematic review and meta-analysis

**DOI:** 10.1097/MD.0000000000032543

**Published:** 2023-01-06

**Authors:** Lingling Guo, Liang Li, Xiuli Li, Linghan Li, Lijin Zhang, Haixia Zhang

**Affiliations:** a Osteopathy Department of Integrated Traditional Chinese and Western Medicine, Zibo Central Hospital, Zibo, Shandong, China; b Department of Chinese Medicine, Zibo Central Hospital, Zibo, Shandong, China; c Department of Plastic and Cosmetic Surgery, Zibo Central Hospital, Zibo, Shandong, China; d Department of Internet, Zibo Central Hospital, Zibo, Shandong, China.

**Keywords:** meta-analysis, pain control, protocol, review, sciatica, warm acupuncture

## Abstract

There are more and more clinical studies on the efficacy of warm acupuncture in treating sciatica, but the systematic review of the efficacy of warm acupuncture is still lacking. The objective of this study was to evaluate the efficacy and safety of warm acupuncture combined with conventional Western medicine in the treatment of sciatica.

**Methods:**

The review was registered in the PROSPERO database (CRD42022379486) and was conducted according to the Preferred Reporting Items for Systematic reviews and Meta-Analyses guidelines. The following databases will be used to search the existing literature (from inception to January 2023): Web of Science, Embase, Cochrane Library, PubMed, Cumulative Index to Nursing and Allied Health Literature, Wanfang, Allied and Complementary Medicine Database, China Knowledge Network, and SPORT-Discus. Included studies are considered eligible if they met the population, intervention, comparator, outcomes, and study design criteria as follows: Population: patients with newly diagnosed sciatica; Intervention: warm acupuncture combined with conventional Western medicine; Comparator: Western medicine alone; Outcomes: total response rate, pain score and pain threshold, adverse events and recurrence rate. Study design: randomized controlled trials or non-randomized cohort studies. Review Manager (RevMan) V.5.3 will be used to perform statistical analyses and to generate forest plots.

**Results:**

The review will add to the existing literature by showing compelling evidence and improved guidance in clinic settings.

## 1. Introduction

Sciatica is a syndrome characterized by neurogenic leg pain, with or without sensory deficits, radiating along the distribution of the sciatic nerve. It is mainly caused by mechanical compression and inflammation following lumbar disc herniation and spinal stenosis. Cytokines such as interleukin 6, interleukin 8 and tumor necrosis factor-α at the site of inflammation may contribute to sciatica.^[1–3]^ Despite the debilitating physical burden of sciatica, its prognosis is good due to its self-limiting nature. However, the diagnosis and treatment of sciatica varies widely within and between countries, which may reflect treatment availability, clinician preference, and socioeconomic variables rather than evidence-based practice.^[4]^

Clinical practice guidelines and systematic reviews recommend several conservative therapies as initial treatment for patients with sciatica.^[5–7]^ Commonly used conservative treatments in clinical practice include bed rest, acupuncture, physical therapy, nonsteroidal anti-inflammatory analgesics, nerve-nourishing drugs, hormones, and even opioids. Considering the public concern about opioid abuse and the favorable safety profile of acupuncture treatment, more and more interest is focused on acupuncture therapy in Chinese medicine. As an important part of Chinese medicine, acupuncture is simple, effective, lightweight, and convenient, and can provide quick pain relief to patients. Warm acupuncture, refers to the moxa roll or moxa cone pointing to the heat of combustion. It is assumed that the steady and deep heat transfer introduced by the inserted needles will exert an effective anti-inflammatory effect at the local lesion, resulting in a strong analgesic effect.^[8–10]^

There are more and more clinical studies on the efficacy of warm acupuncture in treating sciatica, but the systematic review of the efficacy of warm acupuncture is still lacking. The objective of this study was to evaluate the efficacy and safety of warm acupuncture combined with conventional Western medicine in the treatment of sciatica.

## 2. Materials and Methods

### 2.1. Searching strategy

**Figure 1.**
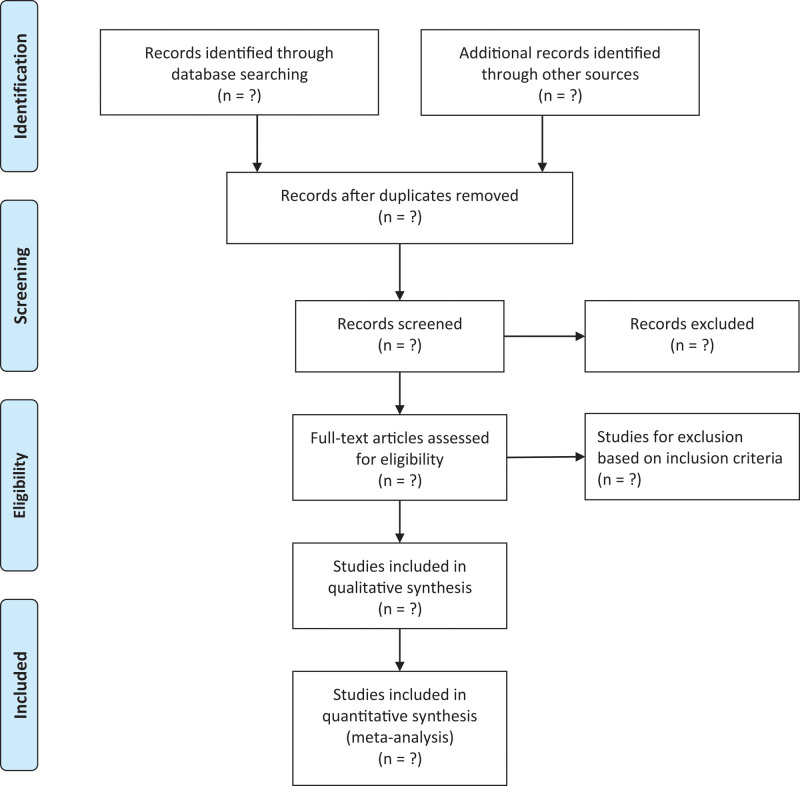
Search strategy for PubMed.

The review was registered in the PROSPERO database (CRD42022379486) and was conducted according to the Preferred Reporting Items for Systematic reviews and Meta-Analyses guidelines. The following databases will be used to search the existing literature (from inception to January 2023): Web of Science, Embase, Cochrane Library, PubMed, Cumulative Index to Nursing and Allied Health Literature, Wanfang, Allied and Complementary Medicine Database, China Knowledge Network, and SPORT-Discus. Reference lists of included studies will be checked for additional studies that are not identified in the database search (Fig. 1). A manual search of abstracts presented at national and international conferences over the past 3 years will be conducted to identify recent unpublished studies. Ethical approval is not required because the data included in the current meta-analysis will be derived from published studies.

### 2.2. Eligibility criteria

Included studies are considered eligible if they met the population, intervention, comparator, outcomes, and study design criteria as follows: Population: patients with newly diagnosed sciatica; Intervention: warm acupuncture combined with conventional Western medicine; Comparator: Western medicine alone; Outcomes: total response rate, pain score and pain threshold, adverse events, and recurrence rate. Study design: randomized controlled trials or non-randomized cohort studies. Exclusion criteria include non-cohort studies, studies with a sample size <50, and studies with insufficient outcome data.

### 2.3. Study selection criteria and selection process

Each title and abstract will be independently screened by pairs of researchers against established criteria. The full text will be independently screened by 2 authors. Disagreements will be resolved by consensus, and if consensus cannot be reached, a third author will be consulted.

### 2.4. Data extraction

The lead author will extract data on the characteristics of the individual trials and all outcomes at all time points into spreadsheets. A second author will check the accuracy of the data. The primary outcomes considered in this review are total response rate, pain score and pain threshold. Secondary outcomes include adverse events and recurrence rate. Outcomes will be extracted from the longest available follow-up (for the primary analysis) and the first available time point after the end of the intervention period (for the sensitivity analysis). For all outcomes, we define a priori a hierarchy of outcome measures based on literature and theoretical considerations and extract data accordingly. When a study reported multiple scales for a given outcome, the highest one of the pain hierarchy scales is selected.

### 2.5. Statistical analysis and data synthesis

Review Manager (RevMan) V.5.3 will be used to perform statistical analyses and to generate forest plots (Copenhagen: The Nordic Cochrane Centre, The Cochrane Collaboration, 2014). A random-effects model will be applied if substantial heterogeneity is found, otherwise, a fixed-effects model will be used. The meta-analysis will be performed using the Mantel–Haenszel method. Sensitivity analyses will be undertaken to determine if the outcome is affected by different classification methods from individual studies. Additionally, publication bias will be assessed using the Begg adjusted rank correlation test and Egger regression asymmetry test. All *P* values will be 2-tailed, and the statistical significance will be set at 0.05.

### 2.6. Risk of bias and quality assessment

The Downs and Black checklist will be used to assess the methodological quality of the included studies.^[11]^ The Cochrane Manual recommends the use of the Downs and Black checklist to assess non-randomized studies. It contains 27 yes/no questions with a total score of 30. Scores are distributed across 5 sections: study quality, external validity, study bias, confounding and selection bias, and power of the study. For randomized controlled trials, the Cochrane Collaboration’s tool will be used to assess the risk of bias in each article.^[12]^ Each article is graded (unclear, low or high risk of bias) according to sequence generation, allocation concealment, blinding of participants and personnel, blinding of outcome assessment, incomplete outcome data, other possible biases, intention-to-treat analysis, selective reporting, and baseline characteristics. Risk of bias assessment is completed by 1 author and checked by a second author. Disagreements are resolved by discussion, and if consensus cannot be reached, a third author will be consulted.

### 2.7. Heterogeneity assessment

Clinical and methodological heterogeneity will be examined and reported for all included studies. Methodological heterogeneity is reported in the pooled table. Statistical heterogeneity in the studies will be assessed by visual analysis of forest plots and the *I*^2^ test. The *I*^2^ test will assess the percentage of inconsistency due to heterogeneity, and values >50% are considered substantial heterogeneity.

## 3. Discussion

Conservative treatment remains the mainstay of treatment for primary sciatica. Some patients have undergone surgical treatment with significant short-term efficacy, but surgery may compromise the stability of the spine. Warm acupuncture therapy is a new method of treating the disease developed on the basis of traditional acupuncture therapy, which has the effects of warming and dispersing cold, resolving phlegm and stasis, reducing swelling and pain, and unblocking the meridians. There are more and more clinical studies on the efficacy of warm acupuncture in treating sciatica, but the systematic review of the efficacy of warm acupuncture is still lacking. The objective of this study was to evaluate the efficacy and safety of warm acupuncture combined with conventional Western medicine in the treatment of sciatica.

## Author contributions

**Conceptualization:** Xiuli Li.

**Data curation:** Lingling Guo, Liang Li, Xiuli Li.

**Formal analysis:** Lingling Guo, Liang Li, Lijin Zhang.

**Funding acquisition:** Lijin Zhang, Haixia Zhang.

**Investigation:** Lingling Guo, Liang Li, Xiuli Li.

**Methodology:** Xiuli Li, Linghan Li.

**Project administration:** Lijin Zhang.

**Software:** Xiuli Li.

**Supervision:** Haixia Zhang.

**Resources:** Lijin Zhang.

**Validation:** Linghan Li.

**Visualization:** Linghan Li.

**Writing – original draft:** Lingling Guo.

**Writing – review & editing:** Linghan Li, Lijin Zhang, Haixia Zhang.
